# MRI-Based Radiomics to Differentiate between Benign and Malignant Parotid Tumors With External Validation

**DOI:** 10.3389/fonc.2021.656918

**Published:** 2021-04-27

**Authors:** Francesca Piludu, Simona Marzi, Marco Ravanelli, Raul Pellini, Renato Covello, Irene Terrenato, Davide Farina, Riccardo Campora, Valentina Ferrazzoli, Antonello Vidiri

**Affiliations:** ^1^ Radiology and Diagnostic Imaging Department, IRCCS Regina Elena National Cancer Institute, Rome, Italy; ^2^ Medical Physics Laboratory, IRCCS Regina Elena National Cancer Institute, Rome, Italy; ^3^ Department of Radiology, University of Brescia, Brescia, Italy; ^4^ Department of Otolaryngology & Head and Neck Surgery, IRCCS Regina Elena National Cancer Institute, Rome, Italy; ^5^ Department of Pathology, IRCCS Regina Elena National Cancer Institute, Rome, Italy; ^6^ Biostatistics-Scientific Direction, IRCCS Regina Elena National Cancer Institute, Rome, Italy; ^7^ Department of Biomedicine and Prevention, University of Rome “Tor Vergata”, Rome, Italy

**Keywords:** head and neck (H&N) cancer, salivary gland (SG) tumors, radiomics, MRI, DWI

## Abstract

**Background:**

The differentiation between benign and malignant parotid lesions is crucial to defining the treatment plan, which highly depends on the tumor histology. We aimed to evaluate the role of MRI-based radiomics using both T2-weighted (T2-w) images and Apparent Diffusion Coefficient (ADC) maps in the differentiation of parotid lesions, in order to develop predictive models with an external validation cohort.

**Materials and Methods:**

A sample of 69 untreated parotid lesions was evaluated retrospectively, including 37 benign (of which 13 were Warthin’s tumors) and 32 malignant tumors. The patient population was divided into three groups: benign lesions (24 cases), Warthin’s lesions (13 cases), and malignant lesions (32 cases), which were compared in pairs. First- and second-order features were derived for each lesion. Margins and contrast enhancement patterns (CE) were qualitatively assessed. The model with the final feature set was achieved using the support vector machine binary classification algorithm.

**Results:**

Models for discriminating between Warthin’s and malignant tumors, benign and Warthin’s tumors and benign and malignant tumors had an accuracy of 86.7%, 91.9% and 80.4%, respectively. After the feature selection process, four parameters for each model were used, including histogram-based features from ADC and T2-w images, shape-based features and types of margins and/or CE. Comparable accuracies were obtained after validation with the external cohort.

**Conclusions:**

Radiomic analysis of ADC, T2-w images, and qualitative scores evaluating margins and CE allowed us to obtain good to excellent diagnostic accuracies in differentiating parotid lesions, which were confirmed with an external validation cohort.

## Introduction

Salivary gland tumors represent about 3-6% of head and neck tumors, with different incidences among tumor histotypes (1). Imaging is commonly used to determine the anatomic origin of the lesions (superficial vs. deep) and the extent of the tumor, in the differentiation between benign and malignant lesions and in the evaluation of neck nodes. This information is crucial to defining the treatment plan, which highly depends on the histology of the tumor. For example, a superficial parotidectomy is performed in cases of pleomorphic adenomas when sited in the superficial portion of the gland, while a total parotidectomy is performed in cases of malignant tumors, and conservative management is the preferred choice for Warthin’s tumors with low potential for malignancy (2).

Fine needle aspiration cytology with or without ultrasonography is an important technique for the pre-surgical evaluation the salivary gland masses. However, considering the rarity and variety of salivary gland neoplasms, particularly malignant lesions, this technique requires great experience and may be inconclusive due to inadequate samples (1, 2).

Ultrasonography (US) and magnetic resonance imaging (MRI) are useful in the evaluation of parotid gland tumors (3, 4). Morphologic features of the lesion can help to separate benign from malignant lesions, including the shape, margins, signal characteristics on T1-weighted and T2-weighted (T2-w) images, type of contrast enhancement (CE), and perineural spread (4, 5). The apparent diffusion coefficient (ADC) derived from diffusion-weighted imaging (DWI) and the enhancement pattern from dynamic contrast-enhanced MRI have also been demonstrated to improve the ability to discriminate benign and malignant lesions (6).

Although the use of multiparametric imaging has increased in recent years, the results are controversial in regard to the role of morphologic and functional parameters derived from multimodal MRI in the differential diagnosis of parotid gland tumors (4, 5, 7, 8). Some studies indicate that sharp margins do not indicate malignancy (7, 8), while others found that heterogeneous CE cannot be used to distinguish benign from malignant lesions (4). An overlap of the mean ADC values between low-grade malignant lesions and benign lesions has also been described (4, 9).

Radiomics is a rapidly emerging field that was proposed a few years ago to extract mineable quantitative features from medical images such as CT, MR, and PET-CT images *via* dedicated algorithms and methodologies (10). The outputs of these analysis are parametric variables that could be correlated with genomic and clinical parameters, particularly in oncologic applications, which provide a more comprehensive tumor description and improve diagnostic accuracy and clinical predictions (11).

Innumerable radiomic features can be calculated in relation to the shape, pixel intensity histogram, and distribution of pixel intensities inside or in the neighborhood of a region of interest (texture analysis), which are potentially useful in predicting the pathological characteristics, response to treatment, and overall survival (12, 13). The purpose of this study is to evaluate the role of MRI-based radiomic analysis using both T2-w images and ADC maps in the differentiation of parotid lesions, and to develop predictive models with validation using an external patient cohort.

## Materials and Methods

### Patients

This study was approved by the institutional review board and was conducted in accordance with the ethical statements of the Declaration of Helsinki. The requirement for informed consent was waived by the institutional review board. This study involved a retrospective evaluation of MRI examinations of 69 patients with parotid gland lesions, consecutively identified in our Institute between 2015 and 2019.

Histopathology diagnosis was obtained in all cases on surgical specimens, by a pathologist who is dedicated to the evaluation of head and neck tumors and has more than 10 years of experience. The exclusion criteria were: recurrence, unsatisfactory image quality, lesions with diameter <5 mm to avoid bias due to partial volume effects.

All patients underwent pre-treatment MRI studies. The patient group included 41 men and 28 women with an average age of 61.1 ± 14.8 years (range 27-90 years). A total of 69 parotid lesions were evaluated, of which 37 were benign, including 13 (18.8%) Warthin’s tumors and 18 (26.1%) cases of pleomorphic adenoma. The other 32 lesions were malignant. Of the 10 parotid metastases, six were from previous cutaneous squamous cell carcinoma and four from previous cutaneous melanoma. The patient and tumor characteristics are provided in more detail in **Table 1**.

**Table 1:** Patients’ characteristics of training and validation cohort.

Characteristic	Training cohort	External Validation cohort
Patient Number	69	44
Age (years)		
Mean ± standard deviation	61.1 ± 14.8	57.5 ± 15
Sex (male/female)	41/28 (59.4%/40.6%)	26/18 (59.1%/40.9%)
Tumor type, n (%)	69 (100%)	44(100%)
Benign	37 (53.6%)	24 (38.6%)
Pleomorphic adenoma	18 (26,1%)	17 (38.6%)
Basal cell adenoma	2 (2.9%)	-
Adenomyoepithelioma	1 (1.5%)	-
Myoepithelial	2 (2.9%)	-
Oncocytoma	1 (1.5%)	-
Warthin tumor	13 (18,8%)	7 (15.9%)
Malignant	32 (36.4%)	20 (45.5%)
Mucoepidermoid carcinoma	5 (7.2%)	3
Acinic cell carcinoma	2 (2.9%)	3
Ductal carcinoma	4 (5.8%)	3
Adenoidocystic carcinoma	6 (8.7%)	4
Lymphoepithelial carcinoma	3 (4.4%)	7 others (3 high grade, 1 cystadenocarcinoma, 3 myoepithelial)
Carcinoma ex pleomorphic adenoma	1 (1.5%)	
Squamous cell carcinoma	1 (1.5%)	
Metastasis	10 (14.5%)	

This population was used as a training cohort and divided into three groups: benign tumors with the exclusion of Warthin’s tumors (24 cases), Warthin’s tumors (13 cases), and malignant tumors (32 cases). Three predictive models were built to compare these groups in pairs. Furthermore, another 44 patients were recruited at the Department of Radiology of the University of Brescia (Italy) and used as an external validation cohort. The tumor characteristics of this cohort are reported in **Table 1**.

### MRI Acquisition Protocols

In Rome, for the training cohort, MRI was performed on a 1.5-T system (Optima MR 450w, GE Health-care, Milwaukee, WI, USA) with dedicated 16-channel receive-only radiofrequency coils: a head coil, a surface neck coil, and a spine coil. The MRI examination included fast spin-eco (FSE) T2-weighted images on the coronal plane (acquisition matrix 288 × 256, field of view 27 x 27 cm, TR/TE 5901 ms/102, slice thickness 4 mm). Next, axial FSE T2-weighted images were obtained (TR/TE 6844 ms/105 ms, field of view 26 cm, in-plane spatial resolution 0.47 mm × 0.47 mm, slice thickness 3 mm, spacing between slices 3.3 mm) along with pre-contrast T1-weighted images (acquisition matrix 288 × 256, field of view 20 cm, TR/TE 617 ms/8.1, slice thickness 3 mm) on the axial plane, which were acquired from the level of the skull base to the thoracic inlet.

DWI was obtained *via* single-shot spin-echo and echo-planar imaging (field of view 26–28 cm, in-plane spatial resolution 2-2.2 mm × 2-2.2 mm TR/TE 4500 ms/77 ms, slice thickness 4 mm, spacing between slices 5 mm, bandwidth 1953 Hz/pixel). Three different b values were used (b = 0, 500, and 800 s/mm^2^) with diffusion-sensitizing gradients applied in three orthogonal directions to obtain trace-weighted images. ADC maps of the training set were generated using the commercial software package Ready View (GE Advantage Workstation, READYView, Palo Alto, CA, USA). The imaging protocol also included post-contrast (Gadolinium 0.1 mmol/kg) T1-w images with liver acquisition with volume acceleration (LAVA) sequences (acquisition matrix 288 × 288, field of view 26-26 cm, TR/TE 9.8 ms/min, slice thickness 1 mm, 214 slices) in axial and coronal planes as required for the routine examination.

In Brescia, for the validation cohort, MRI was performed on a 1.5-T system (Aera, SIEMENS Healthineers Medical Solutions, Knoxville, TN, USA) with dedicated head and neck coils. The parameters of T2-w images were similar to those used for the training cohort (TR/TE 52 20 ms/105 ms, in-plane spatial resolution 0.43 mm × 0.43 mm, slice thickness 3 mm, spacing between slices 4.5 mm). DWI was obtained *via* single-shot spin-echo and echo-planar imaging (field of view, 25 cm in-plane spatial resolution 1.8 mm × 1.8 mm, TR/TE 3900 ms/60 ms, bandwidth 1455 Hz/pixel, slice thickness 3 mm, spacing between slices 4.5 mm). Two different b values were used (b = 50 and 800 s/mm^2^) with diffusion-sensitizing gradients applied in three orthogonal directions to obtain trace-weighted images. ADC maps of the validation cohort were automatically generated by the software MR Syngo (SIEMENS, Healthineers Medical Solutions).

### Extraction of Radiomic Features

The extraction of the radiomic features was performed using S-IBEX software (14). S-IBEX is a standardized version of IBEX (image biomarker explorer) software (15) that was recently adapted and validated according to the guidelines of the Image Biomarker Standardization Initiative (IBSI) (16). The entire tumor volume was delineated by consensus between two radiologists with more than 20 and 10 years of experience in head and neck (A.V. and F.P) using T2-w images.

First- and second-order features were derived from a volumetric analysis of T2-w images, including morphological features (29 features), intensity histogram features (23 features), intensity-volume histogram features (7 features), and grey level co-occurrence matrix or GLCM (25 features). Only first-order features from the intensity direct analysis (9 features) were extracted from ADC maps for a total of 93 features for each lesion. The IBSI reference manual (16) suggests not using some morphological features because they do not have reference values (i.e., the minimum volume enclosing ellipsoid volume and area density, as well as the oriented minimum bounding box volume and area density). Thus, these four features were not included in the statistical analyses, leaving a total of 89 features that were finally evaluated for each lesion.

A description of each feature family is reported in **Supplemental Data**. The formulas used for the calculation are described the IBSI reference manual (16). Details on the image pre-processing, including interpolation, re-segmentation and intensity discretization, are indicated in **Supplemental Data**.

### Qualitative Evaluation of Margins and Contrast Enhancement Type

Two radiologists who have more than 10 years of experience in head and neck and were unaware of the pathological results examined all pre-surgery MRI examinations in relation to the type of margins (regular if the lesion border was well-defined in any sequence or irregular if the lesion border was ill-defined) on both T2- and T1-w images, and the type of CE (1 homogeneous, 2 inhomogeneous, 3 absent) in post contrast T1-w images. The results were obtained by establishing a consensus between the radiologists. The qualitative scores were also included in the feature selection and model building.

### Statistical Analysis

The feature selection and modeling were performed in the Matlab environment. The relationships between categorical variables (type of CE and margins) and the classification response were evaluated using the chi-squared test. The initial selection of the most significant features was carried out using the Mann-Whitney test with a cutoff for p of 0.10. Before further selection of the remaining features, the training and validation datasets were standardized using the z-score normalization method as indicated by Haga et al. (17). Based on this method, each feature was normalized as z=(x-)/std, where x, and std are the feature value, mean value, and standard deviation, respectively. Thus, a *neighborhood component analysis (NCA)* was applied through the Matlab function *fscnca* to further reduce the number of significant variables. To perform NCA, the regularization parameter lambda was tuned to find the optimal lambda value that produces the best classification performance.

In the case of high correlation between the selected features (Spearman correlation coefficient Rho >0.7, p <0.05), the one with the highest accuracy was chosen. The model with the final feature set was achieved using the support vector machine (SVM) binary classification algorithm. A five-fold cross-validation was applied to avoid overfitting due to the small dataset. The classification performance is reported in terms of the accuracy, sensitivity, specificity, positive predictive value (PPV), and negative predictive value (NPV).

## Results

The volumes of benign, Warthin’s, and malignant lesions were 2.7 cm^3^ (range, 0.2-21.1 cm^3^), 5.2 cm^3^ (range, 0.6-69.2 cm^3^), and 5.1 cm^3^ (range, 0.5-114 cm^3^), respectively.

Relevant features included in the predictive models are reported in **Table 2**. The predictive performance of the three models on the training cohort and those tested on the validation cohort is reported in **Tables 3** and **4**, respectively. In the training cohort, the model for discriminating between Warthin’s and malignant tumors reached the best accuracy of 86.7% (sensitivity 87.5%, specificity 84.6%) with a combination of four parameters: the 25^th^ percentile of ADC (P25), the morphological feature of the volume density of the approximate enclosing ellipsoid (AEE) from T2-w images, and the type of margins and enhancement. When this model was tested on the validation cohort, it produced an accuracy of 77.8% (sensitivity 90%, specificity 42.9%).

**Table 2:** Relevant features included in the predictive models.

	Warthin’s Tumors		Malignant Tumors		P value*
	*Median*	*IQR*	*Median*	*IQR*	
P25 of ADC (× 10^-6^ mm^2^/s)	911	190	1058	379	0.054
Volume Density AEE	1.29	0.07	1.26	0.10	0.011
					
	Benign Tumors		Warthin’s Tumors		
	*Median*	*IQR*	*Median*	*IQR*	P value*
P25 of ADC (× 10^-6^ mm^2^/s)	1506.88	612.00	911.00	189.75	<0.001
Volume Density AEE	1.26	0.07	1.29	0.07	0.0481
Minimum Histogram Gradient	-7.25	15.25	-16.00	18.63	0.0582
					
	Benign Tumors		Malignant Tumors		
	*Median*	*IQR*	*Median*	*IQR*	P value*
P25 of ADC (× 10^-6^ mm^2^/s)	1507	612	1058	379	<0.001
P10 of T2	9.00	3.00	6.50	4.00	0.007
					

*P values refer to Mann-Whitney test. P25, 25^th^ percentile of the ADC distribution inside the lesion; P10 of T2, 10^th^ percentile of the T2-weighetd signal intensity distribution inside the lesion; AEE, approximate enclosing ellipsoid.

**Table 3:** Predictive Performance of the three models on the training cohort.

End-point	Selected Features	Accuracy(%)	Sensitivity(%)	Specificity(%)	PPV(%)	NPV(%)
**Warthin’s versus Malignant Tumors**	*ADC P25* *Volume Density AEE* Margins Gd	86.7 [73.2, 95.0]	87.5 [71.0, 96.5]	84.6 [54.5,98.1]	93.3 [79.5, 98.1]	73.3 [51.7, 87.6]
Benign* versus Warthin’s Tumors	*ADC P25* *Volume Density AEE* *MinimumHistogramGradient* Gd	91.9 [78.1, 98.3]	84.6 [54.6, 98.1]	95.8 [78.9, 99.9]	91.7 [61.4, 98.7]	92.0 [76.2, 97.6]
Benign* versus Malignant Tumors	*ADC P25* *T2 P10* Gd Margins	80.4 [67.6, 89.8]	84.4 [67.2, 94.7]	75.0 [53.3, 90.2]	81.8 [68.9, 90.1]	78.2 [60.9, 89.3]

*Benign tumors with exclusion of Warthin’s tumors. Abbreviations as in previous tables. In squared brackets the 95% confidence interval is reported.

**Table 4:** Predictive Performance of the three models tested on the validation cohort.

End-point	Selected Features	Accuracy(%)	Sensitivity(%)	Specificity(%)	PPV(%)	NPV(%)
**Warthin’s versus Malignant Tumors**	*ADC P25* *Volume Density AEE* Margins Gd	81.5 [61.9,93.7]	90.0 [68.3,98.8]	57.1 [18.4,90.1]	85.7 [71.6,93.5]	66.7 [31.7, 89.6]
Benign* versus Warthin’s Tumors	*ADC P25* *Volume Density AEE* *MinimumHistogramGradient* Gd	91.7 [73.0,99.0]	85.7 [42.1,99.6]	94.1 [71.3, 99.9]	85.7 [46.7,97.6]	94.1 [72.2, 99.9]
Benign* versus Malignant Tumors	*ADC P25* *T2 P10* Gd Margins	89.2 [74.6,97.0]	85.0 [62.1,96.8]	94.1 [71.3,99.9]	94.4 [71.6,99.1]	84.2 [65.1, 93.8]

*Benign tumors with exclusion of Warthin’s tumors. Abbreviations as in previous tables. In squared brackets the 95% confidence interval is reported.

In the training cohort, the model for discriminating between benign and Warthin’s tumors showed a high accuracy of 91.9% (sensitivity 84.6%, specificity 95.8%) with a combination of four parameters: P25 of ADC, volume density AEE, minimum histogram gradient from T2-w images, and the type of enhancement. When this model tested on the validation cohort, it produced a comparable accuracy of 91.7% (sensitivity 85.7%, specificity 94.1%).

In the training cohort, the model for discriminating between benign tumors and malignant tumors had an accuracy of 80.4% (sensitivity 84.4%, specificity 75%) with a combination of four parameters: P25 of ADC, P10 from T2-w images, and the types of margins and of CE. When this model was tested on the validation cohort, it produced an accuracy of 89.2% (sensitivity 85%, specificity 94.1%).

**Figure 1:**
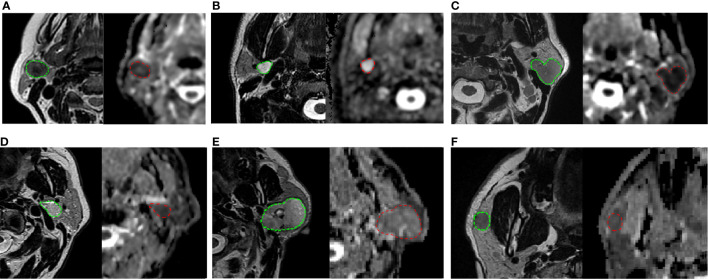
On the top: three correctly classified lesions in the training dataset: **(A)** Warthin’s tumor with low T2 intensity, ovoidal shape and decreased ADC value (P25 of ADC = 0.834 × 10^-3^ mm^2^/s), **(B)** pleomorphic adenoma with typical T2 hyperintensity, sharp margins and high ADC value (P25 of ADC is 1.693 × 10^-3^ mm^2^/s) **(C)** malignant tumor with irregular margins, T2 hypointensity and low ADC value, (P25 = 0.744 × 10^-3^ mm^2^/s). At the bottom: three misdiagnosed cases in the validation set: **(D)** Warthin’s tumor with high T2 hyperintensity and irregular shape (P25 of ADC = 0.930 × 10^-3^ mm^2^/s); **(E)** pleomorphic adenoma with no typical T2 intensity and low ADC value (P25 of ADC = 1.109 × 10^-3^ mm^2^/s); **(F)** malignant tumor with typical very low T2 intensity but regular and sharp margin and ovoidal shape (P25 of ADC = 0.836 × 10^-3^ mm^2^/s). Each frame illustrates T2-weighted axial image with the user-defined lesion contour on the left and the corresponding ADC map on the right.

The results of chi-squared tests performed on the qualitative variables (type of margins and type of CE) included in the model building are reported in **Table 5**. **Figure 1** shows three correct classified lesions in the training dataset and three misdiagnosed cases in the validation set (a Warthin’s tumor, a pleomorphic adenoma, and a malignant tumor, respectively).

**Table 5:** Chi-square Test performed on qualitative variables, Type of Margins (a) and Type of Contrast Enhancement (b) in the three patient groups.

a.				
Type of Enhancement	Homogeneous	Inhomogeneous	Absent	P value
**Warthin’s Tumors**	0	7	6 (28.9%)	0.151
**Malignant Tumors**	1	25	6 (71.1%)	
	(2.2%)	(71.1%)	(26.7%)	
**Benign Tumors**	11	12	1 (64.9%)	0.001
**Warthin’s Tumors**	0	7	6 (35.1%)	
	(29.7%)	(51.4%)	(18.9%)	
**Benign Tumors**	11	12	1 (42.9%)	0.0004
**Malignant Tumors**	1	25	6 (57.1%)	
	(21.4%)	(66.1%)	(12.5%)	

b.			
Type of Margins	Irregular Margins	Regular Margins	P value
**Warthins’ Tumors**	0	13 (28.9%)	0.009
**Malignant Tumors**	19	13 (71.1%)	
	(42.2%)	(57.8%)	
**Benign Tumors**	4	20 (64.9%)	0.315
**Warthins’ Tumors**	0	13 (35.1%)	
	(10.8%)	(89.2%)	
**Benign Tumors**	4	20 (42.9%)	0.003
**Malignant Tumors**	19	13 (57.1%)	
	(41.1%)	(58.9%)	

The values of the most significant features initially selected by the Mann-Whitney test for each group and box plots of the features finally included in the models are shown in **Supplemental Data**.

## Discussion

In the evaluation of parotid gland tumors, there is overlap of the imaging signs between different neoplastic histologies (6, 8, 18), which represents a major limitation in the pre-surgical work-up of these lesions. Some recent studies report that texture analysis of MRI may provide a useful and objective description of signal patterns, which contribute to accurate diagnosis between tumors that look alike by a visual inspection (11, 13). The value of a computer-assisted discrimination of benign and malignant tumors has been explored in various organs (19–21), but only a few studies have assessed the contribution in parotid masses (12, 22–26).

In the present investigation, we identified the most discriminative features from pre-surgery MRI examinations based on first- and second-order texture analyses of T2-w images and first-order texture analysis of ADC maps for the separation of benign and malignant parotid lesions. All the three proposed models had good to excellent predictive performance, in combination with qualitative scores related to the type of margins or CE. This suggests that the texture analysis should be used as an additional tool for supporting radiologists’ decisions and not in isolation (22).

Consistent with prior studies, we found significantly lower ADC values for Warthin’s tumors than those of benign and malignant tumors (27, 28). Among the ADC-derived parameters, the 25^th^ percentile (P25) of the ADC distribution inside the lesion was found to be the most relevant and was selected in all three models. This confirms the important role of DWI for the differential diagnosis of parotid lesions, as reported in previous studies (4, 9, 22, 29, 30). The P25 of ADC represents the ADC value associated with the tumor sub-volume with the most restrictive water molecule mobility. Thus, it is potentially related to a tumor region with a higher cell density. This finding suggests that instead of mean/median ADC values, it would be preferable to use a histogram-based approach to better address the tissue heterogeneity inside the tumor, which typically characterizes both benign and malignant parotid lesions (6, 8, 18). In this context, an interesting study of Khalek Abdel Razek et al. (31) evaluated the added value of Diffusion Tensor Imaging (DTI) to differentiate subtypes of parotid tumors, on the basis of fractional anisotropy (FA) and mean diffusivity, reporting very high accuracies. Even though DTI cannot be considered part of routine head and neck oncologic protocols, it showed a great potential to accurately separate Warthin’s tumors from malignant tumors, as well as Warthin’s tumours among all the other benign tumors. In particular, FA appeared to be associated to the complexity and heterogeneity of tissue microstructure and it may provide deeper insights into the parotid tumor cytoarchitecture, compared to conventional ADC.

The volume density AEE derived from T2-w images showed discriminatory potential for separating Warthin’s tumors from other parotid tumors. This feature is directly related to the volume sphericity and showed increased values for Warthins’ tumors, indicating that this kind of lesion has a more spherical shape than both malignant and benign tumors (p = 0.025 and p = 0.04 respectively, Mann-Whitney test). Concerning T2-w images, P10 was found to be relevant for differentiating benign and malignant lesions, with the latter showing significantly lower P10 values (p = 0.007, Mann-Whitney test) according to previous studies (5, 19, 23, 32, 33). In fact, it was demonstrated that high, intermediate, and low signal intensity can be associated with benign lesions (pleomorphic adenoma), intermediate, and highly malignant tumors, respectively (5, 32). Therefore, the use of heavily T2-w sequences is strongly suggested (33).

Recently, Sarioglu et al. (12) reported a texture-based study of T2-w images and contrast-enhanced T1-w images to discriminate the most common parotid tumors, in addition to several qualitative scores, as we similarly proposed. Due to the small number of malignant parotid tumors included and to differences in MR sequences considered for the analysis, a direct comparison with our findings is not possible. However, the authors showed that both skewness and kurtosis were significantly different between pleomorphic adenoma, Warthin’s tumors, and mucoepidermoid carcinoma. In the present study, the skewness was also found to be discriminative for the separation of benign and malignant/Warthin’s lesions, even though it was not included in the final model. The role of the minimum histogram gradient from T2-w images in the model is less obvious for differentiating benign and Warthin’s tumors. This feature was strongly associated with several GLCM-based features, such as the dissimilarity, contrast, and inverse difference (Spearman’s coefficient Rho = 0.894, 0.870, and -0.906 with p <0.0001), with the latter being a measure of homogeneity (16). Therefore, an increased value of the minimum histogram gradient in the group of benign lesions should suggest higher contrast and inhomogeneity compared to the group of Warthin’s tumors.

The explanation for this phenomenon is not straightforward, considering that Warthin’s lesions typically show high tissue heterogeneity (22, 27). In fact, the tissue contrast of this kind of lesion can be affected by degenerative alterations in the interstitial tissue and may depend on the degree of differentiation of tumor cells, as well as the presence or absence of necrosis and cystic components (27, 34). This causes a broad range of MR signal intensity, which reflects the variable proportion of microcytic components and lymphoid stroma inside the lesion (34). On the other hand, benign lesions may also show tissue heterogeneity on T2-w images due to cystic, solid, or mixoid components. Moreover, it was recently reported that the volume of the lesion may impact the value of some T2-w radiomic features, such as dissimilarity and energy, as shown by Wormald et al. (35). They found that larger cervical cancers had lower dissimilarity and higher energy and thus higher homogeneity and uniformity than smaller ones. In our dataset, Warthin’s tumors showed a tendency to be larger (median, 5.2 cm^3^) than benign lesions (median, 2.7 cm^3^), even though there was no statistically significant difference between these volumes (p = 0.22, Mann-Whitney test). This may partially explain our findings.

The macroscopic imaging signs involved in the models (i.e., the type of margins and CE) were previously found to be useful in differential diagnosis (12). In fact, an ill-defined tumor border and low-grade contrast enhancement were observed as independent risk factors for malignancy, while a well-defined tumor margin was reported as a good qualitative indicator of benignity (12, 23).

The potential role of contrast enhancement and perfusion in discriminating various subtypes of parotid tumors was specifically addressed by some previous studies, which proposed the use of arterial-spin labeling (ASL) (30), dynamic susceptibility contrast perfusion-weighted MRI or dynamic contrast enhanced MRI (2, 4, 36, 37). These investigations consistently indicated that Warthins’s tumors are characterized by a higher tissue vascularity than pleomorphic adenomas, and generally by a lower vascularity than malignant tumors. Although further efforts should be made to improve the repeatability and reproducibility of perfusion-weighted techniques (38) before including them as part of routine head and neck oncologic protocols, they are very promising and merit future investigation.

Lastly, we tested the performance of the developed prediction models on an external validation dataset, which is strongly suggested for a complete radiomic analysis to verify the reproducibility and transportability in a clinical setting (10). The predictive performance on the validation cohort indicated comparable accuracies, even though the model discriminating between Warthin’s and malignant tumors showed lower specificity and negative predictive power (they decreased from 86.6 to 57.1, and from 73.3 to 66.7%, respectively).

This is consistent with a recent study of Gabelloni et al. (25), who also proposed a radiomic analysis of parotid tumors on T2-w MR images and obtained the best classification performance when comparing benign tumors with Warthin’s tumors, while a lower accuracy was found in differentiating Warthin’s and malignant tumors. Radiologists have particular difficulties in differentiating this type of lesion, the reason is that it may present a solid component with low signal intensity in T2-w images, which is also found in malignant lesions. Furthermore, they may have cystic components with low and high signal intensity in T1-w images, which indicate the presence of cystic fluid or high protein fluid, respectively (2, 27, 39). As mentioned, DWI has the potential to appreciably improve this misclassification, as Warthin’s tumors typically show lower ADC values than both benign and low grade malignant tumors (22, 27).

Recent literature has shown a growing interest in the clinical applicability of radiomics for the parotid tumor characterization (12, 25, 26), thanks to significant improvements in diagnostic accuracy obtained with a multiparametric approach to quantitative MRI (2, 4, 6, 30, 31, 36, 37). However, no consensus exists regarding the most appropriate sequences to consider for the extraction of radiomics features and only a few studies used standardized software, previously validated according to the updated IBSI guidelines (16). Moreover, the lack of an external validation set in most of current papers makes impossible to verify the transportability of the proposed models. In general, further efforts are needed for a standardization of the entire workflow, from image acquisition and processing to feature extraction, statistical analysis and clinical validation (40). This could facilitate a direct comparison between findings from single centers, helping to clarify the added role of MRI-based radiomics in oncologic applications.

The present study has some limitations. First, its retrospective nature may have introduced bias and confounding factors. Secondly, our findings should be confirmed in a larger patient population as only a small number of benign and malignant tumors were included in the training cohort. Another limitation is the lack of differentiation between low and high-grade lesions in the context of malignant neoplasms due to the low number of patients, which could allow us to develop specific predictive models as a function of tumor grade.

## Conclusions

Radiomic analysis of ADC and T2-w images in addition to qualitative scores evaluating margins and CE allowed us to obtain good to excellent diagnostic accuracies in differentiating parotid lesions, which was confirmed by testing on an external validation cohort.

## Data Availability Statement

The original contributions presented in the study are included in the article/**Supplementary Material**. Further inquiries can be directed to the corresponding author.

## Ethics Statement

The studies involving human participants were reviewed and approved by Institutional Review Board, Regina Elena National Cancer Institute (IRCCS), Rome, Italy. Written informed consent for participation was not required for this study in accordance with the national legislation and the institutional requirements.

## Author Contributions

FP and AV conceived of the study and drafted the manuscript. SM carried out the radiomic analysis and contributed to the draft. RP contributed to the patient enrollment and data gathering. RCo provided the histological data on surgical specimens. VF and FP carried out the image delineation and participated in the image analysis. MR, DF, and RCa provided the external validation dataset and contributed to the design of the study. IT performed the statistical analysis. All authors contributed to the article and approved the submitted version. 

## Conflict of Interest

The authors declare that the research was conducted in the absence of any commercial or financial relationships that could be construed as a potential conflict of interest.

## References

[1] Gao M Hao Y Huang MX Ma DQ Chen Y Luo HY. Salivary Gland Tumours in a Northern Chinese Population: A 50-Year Retrospective Study of 7190 Cases. Int J Oral Maxillofac Surg (2017) 46:343–9. 10.1016/j.ijom.2016.09.021 27769738

[2] Espinoza S Felter A Malinvaud D Badoual C Chatellier G Siauve N. Warthin’s Tumor of Parotid Gland: Surgery or Follow-Up? Diagnostic Value of a Decisional Algorithm With Functional MR. Diagn Interventional Imaging (2016) 97:37–43. 10.1016/j.diii.2014.11.024 25543869

[3] Monsour N Hofauer B. Knopf AJ Ultrasound Elastography in Diffuse and Focal Parotid Gland Lesions. Otorhinolaryngol Relat Spec (2017) 79:54–64. 10.1159/000455727 28231589

[4] Yuan Y Tang W Tao X. Parotid Gland Lesions: Separate and Combined Diagnostic Value of Conventional MRI, Diffusion-Weighted Imaging and Dynamic Contrast-Enhanced MRI. Br J Radiol (2016) 89(1060):20150912. 10.1259/bjr.20150912 26892378 PMC4846216

[5] Christe A Waldherr C Hallett R Zbaeren P Thoeny H. MR Imaging of Parotid Tumors: Typical Lesion Characteristics in MR Imaging Improve Discrimination Between Benign and Malignant Disease. AJNR Am J Neuroradiol (2011) 32:1202–7. 10.3174/ajnr.A2520 PMC7966029 21724574

[6] Gökçe E. Multiparametric Magnetic Resonance Imaging for the Diagnosis and Differential Diagnosis of Parotid Gland Tumors. J Magn Reson Imaging (2020) 52:11–32. 10.1002/jmri.27061 32065489

[7] Teresi LM Lufkin RB Wortham DC Abemayor E Hanafee WN. Parotid Masses: MR Imaging. Radiology (1989) 163:405–9. 10.1148/radiology.163.2.3562818 3562818

[8] Kessler AT Bhatt AA. Review of the Major and Minor Salivary Glands, Part 2: Neoplasms and Tumor-like Lesions. J Clin Imaging Sci (2018) 8:48. 10.4103/jcis.JCIS_46_18 30546932 PMC6251244

[9] Habermann CR Arndt C Graessner J Diestel L Petersen KU Reitmeier F. Diffusion-Weighted Echo-Planar MR Imaging of Primary Parotid Gland Tumors: Is a Prediction of Different Histologic Subtypes Possible? AJNR Am J Neuroradiol (2009) 30:591–6. 10.3174/ajnr.A1412 PMC7051445 19131405

[10] Lambin P Leijenaar RTH Deist TM Peerlings J de Jong EEC van Timmeren J. Radiomics: The Bridge Between Medical Imaging and Personalized Medicine. Nat Rev Clin Oncol (2017) 14:749–62. 10.1038/nrclinonc.2017.141 28975929

[11] Song J Yin Y Wang H Zhihui Chang Z Zhaoyu Liu Z Cui L. A Review of Original Articles Published in the Emerging Field of Radiomic. Eur J Radiol (2020) 127:108991. 10.1016/j.ejrad.2020.108991 32334372

[12] Sarioglu O Sarioglu FC Akdogan AI Kucuk U Arslan IB Cukurova I. MRI-Based Texture Analysis to Differentiate the Most Common Parotid Tumours. Clin Radiol (2020) 75:877.e15–e23. 10.1016/j.crad.2020.06.018 32703544

[13] Kassner A Thornhill RE. Texture Analysis: A Review of Neurologic MRI Applications. AJNR Am J Neuroradiol (2010) 31:809–16. 10.3174/ajnr.A2061 PMC7964174 20395383

[14] Bettinelli A Branchini M De Monte F Scaggion A Paiusco M. Technical Note: An IBEX Adaption Toward Image Biomarker Standardization. Med Phys (2020) 47(3):1167–73. 10.1002/mp.13956 31830303

[15] Zhang L Fried DV Fave XJ Hunter LA Yang J Court LE. Ibex: An Open Infrastructure Software Platform to Facilitate Collaborative Work in Radiomics. Med Phys (2015) 42:1341–53. 10.1118/1.4908210 PMC5148126 25735289

[16] Zwanenburg A Leger S Vallières M Löck S. Image Biomarker Standardisation Initiative:Standardized Quantitative Radiomics for High-Throughput Image-based Phenotyping. Radiology (2020) 295:328–38. 10.1148/radiol.2020191145 PMC7193906 32154773

[17] Haga A Takahashi W Aoki S Nawa K Yamashita H Abe O. Standardization of Imaging Features for Radiomics Analysis. J Med Invest (2019) 66:35–7. 10.2152/jmi.66.35 31064950

[18] Abdel Razek AAK Mukherji SK. State-of-the-Art Imaging of Salivary Gland Tumors. Neuroimaging Clin N Am (2018) 28:303–17. 10.1016/j.nic.2018.01.009 29622121

[19] Mayerhoefera ME Breitensehera M Amannd G Dominkuse M. Are Signal Intensity and Homogeneity Useful Parameters for Distinguishing Between Benign and Malignant Soft Tissue Masses on MR Images? Objective Evaluation by Means of Texture Analysis. Magn Reson Imaging (2008) 26:1316–22. 10.1016/j.mri.2008.02.013 18448302

[20] Wang H Zhang J Bao S Liu J Hou F Huang Y. Preoperative MRI-Based Radiomic Machine-Learning Nomogram May Accurately Distinguish Between Benign and Malignant Soft-Tissue Lesions: A Two-Center Study. J Magn Reson Imaging (2020) 52:873–82. 10.1002/jmri.27111 32112598

[21] Nogueira L Nunes RG. Radiomics Based on Multimodal MRI for the Differential Diagnosis of Benign and Malignant Breast Lesions, Editorial. J Magn Reson Imaging (2020) 52:608–9. 10.1002/jmri.27168 32333824

[22] Fruehwald-Pallamar J Czerny C Holzer-Fruehwald L Nemec SF Mueller-Mang C Weber M. Texture-Based and Diffusion-Weighted Discrimination of Parotid Gland Lesions on MR Images At 3.0 Tesla. NMR BioMed (2013) 26:1372–9. 10.1002/nbm.2962 23703801

[23] Wu Q Zhu LN Jiang JS Bu SS Xu XQ Wu FY. Characterization of Parotid Gland Tumors Using T2 Mapping Imaging: Initial Findings. Acta Radiol (2020) 61:629–35. 10.1177/0284185119875646 31542938

[24] Chang YJ Huang TY Liu YJ Chung HW Juan CJ. Classification of Parotid Gland Tumors by Using Multimodal MRI and Deep Learning. NMR BioMed (2020) 34:e4408. 10.1002/nbm.4408 32886955 PMC7757221

[25] Gabelloni M Faggioni L Attanasio S Vani V Goddi A Colantonio S. Can Magnetic Resonance Radiomics Analysis Discriminate Parotid Gland Tumors? A Pilot Study. Diagnostics (Basel) (2020) 310(11):900. 10.3390/diagnostics10110900 PMC7692594 33153140

[26] Zheng YM Li J Liu S Cui JF Zhan JF Pang J. Mri-Based Radiomics Nomogram for Differentiation of Benign and Malignant Lesions of the Parotid Gland. Eur Radiol (2020) 19:1–11. 10.1007/s00330-020-07483-4 33211145

[27] Wang CW Chu YH Chiu DY Shin N Hsu HH Lee JC. Journal CLUB: The Warthin Tumor Score: A Simple and Reliable Method to Distinguish Warthin Tumors From Pleomorphic Adenomas and Carcinomas. AJR Am J Roentgenol (2018) 210:1330–7. 10.2214/AJR.17.18492 29667889

[28] Zhang Z Song C Zhang Y Wen B Zhu J Cheng J. Apparent Diffusion Coefficient (ADC) Histogram Analysis: Differentiation of Benign From Malignant Parotid Gland Tumors Using Readout-Segmented Diffusion-Weighted Imaging. Dentomaxillofac Radiol (2019) 48:20190100. 10.1259/dmfr.20190100 31265331 PMC6775791

[29] Matsushima N Maeda M Takamura M. Takeda K.Apparent Diffusion Coefficients of Benign and Malignant Salivary Gland Tumors. Comparison to Histopathological Findings. J Neuroradiol (2017) 34:183–9. 10.1016/j.neurad.2007.04.002 17568674

[30] Razek AAKA. Multi-Parametric MR Imaging Using Pseudo-Continuous Arterial-Spin Labeling and Diffusion-Weighted MR Imaging in Differentiating Subtypes of Parotid Tumors. Magn Reson Imaging (2019) 63:55–9. 10.1016/j.mri.2019.08.005 31422165

[31] Khalek Abdel Razek AA. Characterization of Salivary Gland Tumours With Diffusion Tensor Imaging. Dentomaxillofac Radiol (2018) 47:20170343. 10.1259/dmfr.20170343 29412748 PMC6196043

[32] Espinoza S Halimi P. Interpretation Pearls For MR Imaging Of Parotid Gland Tumor. Eur Ann Otorhinolaryngol Head Neck Dis (2013) 130:30–5. 10.1016/j.anorl.2011.12.006 22819222

[33] Sakamoto M Sasano T Higano S Takahashi S Iikubo M Kakehata S. Usefulness of Heavily T(2) Weighted Magnetic Resonance Images for the Differential Diagnosis of Parotid Tumours. Dentomaxillofac Radiol (2003) 32:295–9. 10.1259/dmfr/32387150 14709603

[34] Kato H Fujimoto K Matsuo M Mizuta K. Aoki M. Usefulness of Diffusion-Weighted MR Imaging for Differentiating Between Warthin’s Tumor and Oncocytoma of the Parotid Gland. Jpn J Radiol (2017) 35:78–85. 10.1007/s11604-016-0608-5 28074380

[35] Wormald BW Doran SJ Ind TE D’Arcy J Petts J deSouza NM. Radiomic Features of Cervical Cancer on T2- and Diffusion-Weighted MRI: Prognostic Value in Low-Volume Tumors Suitable for Trachelectomy. Gynecol Oncol (2020) 156:107–14. 10.1016/j.ygyno.2019.10.010 PMC7001101 31685232

[36] Razek AAKA. Multi-Parametric MR Imaging Using Pseudo-Continuous Arterial-Spin Labeling and Diffusion-Weighted MR Imaging in Differentiating Subtypes of Parotid Tumors. Magn Reson Imaging (2019) 63:55–9. 10.1016/j.mri.2019.08.005 31422165

[37] Xu Z Zheng S Pan A Cheng X Gao M. A Multiparametric Analysis Based on DCE-MRI to Improve the Accuracy of Parotid Tumor Discrimination. Eur J Nucl Med Mol Imaging (2019) 46:2228–34. 10.1007/s00259-019-04447-9 31372671

[38] Shukla-Dave A Obuchowski NA Chenevert TL Jambawalikar S Schwartz LH Malyarenko D. Quantitative Imaging Biomarkers Alliance (QIBA) Recommendations for Improved Precision of DWI and DCE-MRI Derived Biomarkers in Multicenter Oncology Trials. J Magn Reson Imaging (2019) 49:e101–21. 10.1002/jmri.26518 PMC6526078 30451345

[39] Ikeda M Motoori K Hanazawa T Nagai Y Yamamoto S Ueda T. Warthin Tumor of the Parotid Gland: Diagnostic Value of MR Imaging With Histopathologic Correlation. AJNR Am J Neuroradiol (2004) 25:1256–62. 10.1016/j.ijrobp.2018.08.032 PMC7976549 15313720

[40] Morin O Vallières M Jochems A Woodruff HC Valdes G Braunstein SE. A Deep Look Into the Future of Quantitative Imaging in Oncology: A Statement of Working Principles and Proposal for Change. Int J Radiat Oncol Biol Phys (2018) 102(4):1074–82. 10.1016/j.ijrobp.2018.08.032 30170101

