# Supplementary material for: MRI-Based Radiomics to Differentiate between Benign and Malignant Parotid Tumors With External Validation
Source: Front Oncol. 2021 Apr 27;11:656918. doi: 10.3389/fonc.2021.656918 (PMC8111169; doi:10.3389/fonc.2021.656918)
Supplement: Supplementary file 2 [file DataSheet_2.pdf]

**Supplementary Table 1** List of included Radiomic Features

| Feature Family (count) | | | | |
|---|---|---|---|---|
| **Morphological (29)** | **IntensityHistogram(23)** | **IntensityVolumeHistogram(7)** | **GrayLevelCooccurenceMatrix(25)** | **IntensityDirect(9)** |
| Volume | Mean | VolumeIntFract_10 | JointMaximum | GlobalEntropy |
| ApproximateVolume | Variance | VolumeIntFract_90 | JointAverage | GlobalMean |
| SurfaceArea | Skewness | IntensityVolFract_10 | JointVariance | GlobalMedian |
| SurfaceToVolumeRatio | Kurtosis | IntensityVolFract_90 | JointEntropy | InterQuartileRange |
| Compactness1 | Median | VolumeFractionDiff | DifferenceAverage | Kurtosis |
| Compactness2 | Min | IntensityFractDiff | DifferenceVariance | Skewness |
| SphericalDisproportion | P10 | AreaUnderIVHCurve | DifferenceEntropy | P25 |
| Sphericity | P90 | | SumAverage | P75 |
| Asphericity | Max | | SumVariance | |
| CentreOfMassShift | Mode | | SumEntropy | |
| Maximum3DDiameter | InterQuartileRange | | AngularSecondMoment | |
| MajorAxisLength | Range | | Contrast | |
| MinorAxisLength | MeanAbsoluteDeviation | | Dissimilarity | |
| LeastAxisLength | RobustMeanAbsoluteDeviation | | InverseDifference | |
| Elongation | MedianAbsoluteDeviation | | InverseDiffNorm | |
| Flatness | CoefficientOfVariation | | InverseDiffMoment | |
| VolumeDensity_AABB | QuartileCoefficientOfDispersion | | InverseDiffMomentNorm | |
| AreaDensity_AABB | Entropy | | InverseVariance | |
| VolumeDensity_OMBB | Uniformity | | Correlation | |
| AreaDensity_OMBB | MaximumHistogramGradient | | AutoCorrelation | |
| VolumeDensity_AEE | MaximumHistogramGradientGL | | ClusterTendendcy | |
| AreaDensity_AEE | MinimumHistogramGradient | | ClusterShade | |
| VolumeDensity_MVEE | MinimumHistogramGradientGL | | ClusterProminence | |
| AreaDensity_MVEE | | | InformationMeasureCor1 | |
| VolumeDensity_CH | | | InformationMeasureCor2 | |
| AreaDensity_CH | | | | |
| IntegratedIntensity | | | | |
| MoransIIndex | | | | |
| GearysCMeasure | | | | |

A detailed description of radiomic features included in each family is reported in IBSI reference manual (Zwanenburg A, Leger S, Vallières M, Löck S. Image biomarker standardisation initiative. arXiv preprint arXiv:1612.07003).

**Supplementary Table 2** Pre-processing of Radiomic Features

**T2-weighted Images**

| Feature Family | Count | Interpolation | Re-segmentation | Intensity Discretization |
|---|---|---|---|---|
| Morphology | 29 | - | - | - |
| Intensity Histogram | 23 | 3D (2x2x2 $mm^3$) | - | Fixed Bin Number (32) |
| Intensity Volume Histogram | 7 | 3D (2x2x2 $mm^3$) | - | Fixed Bin Number (32) |
| GrayLevelCooccurenceMatrix* | 25 | 3D (2x2x2 $mm^3$) | - | Fixed Bin Number (64) |

**ADC maps**

| Feature Family | Count | Interpolation | Re-segmentation | Intensity Discretization |
|---|---|---|---|---|
| Intensity Direct | 9 | - | Range: [0.25-3]$\times10^{-3}$ $mm^2/s$ | Fixed Bin Size (0.1$\times10^{-3}$ $mm^2/s$) |

*Aggregation method: 3D average; Directions: [0 1 2 3 4 5 6 7 8 9 10 11 12]. A detailed description of radiomic features included in each family is reported in IBSI reference manual (Zwanenburg A, Leger S, Vallières M, Löck S. Image biomarker standardisation initiative. arXiv preprint arXiv:1612.07003).

**Supplementary Table 3. Values of the most significant features for separation of Warthins' and Malignant Tumors**

| | Warthins' Tumors | | Malignant Tumors | | |
|---|---|---|---|---|---|
| | *Median* | *IQR* | *Median* | *IQR* | *P value** |
| **P25 of ADC($\times 10^{-6}$ $mm^2/s$)** | 911 | 190 | 1058 | 379 | 0.054 |
| **VolumeDensity AEE** | 1.29 | 0.07 | 1.26 | 0.10 | 0.011 |
| **VolumeDensity CH** | 0.82 | 0.09 | 0.78 | 0.12 | 0.025 |

P values refer to Mann-Whitney tes

**Supplementary Table 4. Values of most significant features for differentiating Benign vs Warthins' Tumors**

| | Benign Tumors | | Warthins' Tumor | | |
|---|---|---|---|---|---|
| | *Median* | *IQR* | *Median* | *IQR* | *P value** |
| **Median ADC (× $10^{-6}$ $mm^2/s$)** | 1715,50 | 660,50 | 1069,00 | 324,88 | <0,001 |
| **Skewness ADC (× $10^{-6}$ $mm^2/s$)** | -0,03 | 1,04 | 0,48 | 0,74 | 0,021 |
| **Mean ADC (× $10^{-6}$ $mm^2/s$)** | 1702,93 | 641,20 | 1142,26 | 361,11 | <0,001 |
| **ADC P25 (× $10^{-6}$ $mm^2/s$)** | 1506,88 | 612,00 | 911,00 | 189,75 | <0,001 |
| **ADC P75 (× $10^{-6}$ $mm^2/s$)** | 1859,50 | 692,88 | 1409,25 | 434,19 | 0,001 |
| **Mean T2** | 15,82 | 5,61 | 14,46 | 5,12 | 0,050 |
| **Skewness T2** | -0,16 | 0,91 | 0,78 | 0,94 | 0,040 |
| **Median T2** | 16,00 | 7,00 | 14,00 | 6,00 | 0,080 |
| **P90 of T2** | 23,50 | 7,00 | 21,00 | 8,00 | 0,085 |
| **RobustMeanAbsoluteDeviation** | 3,35 | 1,58 | 2,56 | 1,27 | 0,077 |
| **MaximumHistogramGradient** | 6,50 | 20,25 | 18,00 | 32,13 | 0,065 |
| **MinimumHistogramGradient** | -7,25 | 15,25 | -16,00 | 18,63 | 0,058 |
| **MinimumHistogramGradientGL** | 19,00 | 13,00 | 14,00 | 6,75 | 0,069 |
| **AreaDensity AABB** | 0,62 | 0,02 | 0,66 | 0,08 | 0,019 |
| **VolumeDensity AEE** | 1,26 | 0,07 | 1,29 | 0,07 | 0,048 |
| **VolumeDensity_CH** | 0,78 | 0,06 | 0,82 | 0,09 | 0,061 |
| **AreaUnderIVHCurve** | 0,49 | 0,18 | 0,45 | 0,17 | 0,050 |
| **JointAverage** | 32,64 | 14,15 | 26,90 | 9,58 | 0,023 |
| **DifferenceAverage** | 8,89 | 4,33 | 6,81 | 2,01 | 0,047 |
| **DifferenceVariance** | 64,97 | 47,37 | 40,31 | 23,85 | 0,058 |
| **SumAverage** | 64,56 | 26,82 | 53,80 | 19,16 | 0,021 |
| **Contrast** | 150,54 | 136,31 | 85,25 | 59,18 | 0,067 |
| **Dissimilarity** | 8,89 | 4,33 | 6,81 | 2,01 | 0,047 |
| **InverseDifference** | 0,21 | 0,06 | 0,25 | 0,04 | 0,032 |
| **InverseDiffNorm** | 0,89 | 0,04 | 0,91 | 0,02 | 0,034 |
| **InverseDiffMoment** | 0,13 | 0,05 | 0,16 | 0,04 | 0,034 |
| **InverseDiffMomentNorm** | 0,97 | 0,03 | 0,98 | 0,01 | 0,058 |
| **InverseVariance** | 0,13 | 0,04 | 0,16 | 0,04 | 0,047 |
| **AutoCorrelation** | 1089,46 | 884,45 | 795,49 | 465,05 | 0,025 |
| **ClusterShade** | -838,35 | 4825,44 | 1886,75 | 2160,23 | 0,058 |

* P values refer to Mann-Whitney test

**Supplementary Table 5. Values of most significant features for differentiating Benign vs Malignant Tumors**

| | Benign Tumors | | Malignant Tumors | | |
|---|---|---|---|---|---|
| | *Median* | *IQR* | *Median* | *IQR* | *P value** |
| **median ADC** ($\times 10^{-6}$ $mm^2/s$) | 1716 | 661 | 1179 | 435 | <0,001 |
| **skewness ADC** | -0.03 | 1.04 | 0.69 | 1.06 | 0.001 |
| **mean ADC** ($\times 10^{-6}$ $mm^2/s$) | 1703 | 641 | 1230 | 441 | <0,001 |
| **P25 of ADC** ($\times 10^{-6}$ $mm^2/s$) | 1507 | 612 | 1058 | 379 | <0,001 |
| **P75 of ADC** ($\times 10^{-6}$ $mm^2/s$) | 1860 | 693 | 1358 | 463 | <0,001 |
| **Mean T2** | 15.8 | 5.6 | 12.8 | 4.1 | 0.008 |
| **Skewness T2** | -0.16 | 0.91 | 0.56 | 1.08 | 0.009 |
| **Median T2** | 16.00 | 7.00 | 12.00 | 4.00 | 0.008 |
| **P10 of T2** | 9.00 | 3.00 | 6.50 | 4.00 | 0.007 |
| **P90 of T2** | 23.50 | 7.00 | 20.00 | 9.00 | 0.038 |
| **Mode T2** | 16.0 | 10.0 | 10.5 | 5.0 | 0.012 |
| **MaximumHistogramGradientGL** | 12.0 | 8.5 | 8.0 | 6.0 | 0.052 |
| **MinimumHistogramGradientGL** | 19.00 | 13.00 | 13.00 | 5.50 | 0.009 |
| **AreaDensity AEE** | 1.58 | 0.15 | 1.68 | 0.21 | 0.009 |
| **IntensityVolFract 90** | 10.00 | 3.00 | 7.50 | 4.00 | 0.007 |
| **AreaUnderIVHCurve** | 0.49 | 0.18 | 0.40 | 0.13 | 0.008 |
| **JointAverage** | 32.6 | 14.1 | 25.5 | 9.1 | 0.013 |
| **SumAverage** | 64.6 | 26.8 | 51.1 | 18.3 | 0.012 |
| **AutoCorrelation** | 1089 | 884 | 731 | 486 | 0.013 |
| **ClusterShade** | -838 | 4825 | 1494 | 4187 | 0.024 |

* P values refer to Mann-Whitney test

## Confusion Matrix

| | |
|---|---|
| TP | FP |
| FN | TN |

**TP = true positive; FP = false positive; TN = true negative; FN = false negative;**

1) **Warthin's versus Malignant tumors**

**Training Cohort**

| | |
|---|---|
| 28 | 2 |
| 4 | 11 |

**Validation Cohort**

| | |
|---|---|
| 18 | 3 |
| 2 | 4 |

2) **Benign versus Warthin's tumors**

**Training Cohort**

| | |
|---|---|
| 11 | 1 |
| 2 | 23 |

**Validation Cohort**

| | |
|---|---|
| 6 | 1 |
| 1 | 16 |

3) **Benign versus Malignant tumors**

**Training Cohort**

| | |
|---|---|
| 27 | 6 |
| 5 | 18 |

**Validation Cohort**

| | |
|---|---|
| 17 | 1 |
| 3 | 16 |

**Supplementary Figures:**

**Box-and-whisker plots of the relevant features included in the models**

**Figure 1.** Box-and-whisker plots of the two features included in the predictive model for discriminating Warthin's versus malignant tumors: the 25th percentile (P25) of ADC and the morphological feature Volume Density AEE (Approximate Enclosing Ellipsoid) from T2-w images. P-values refer to the Mann-Whitney test.

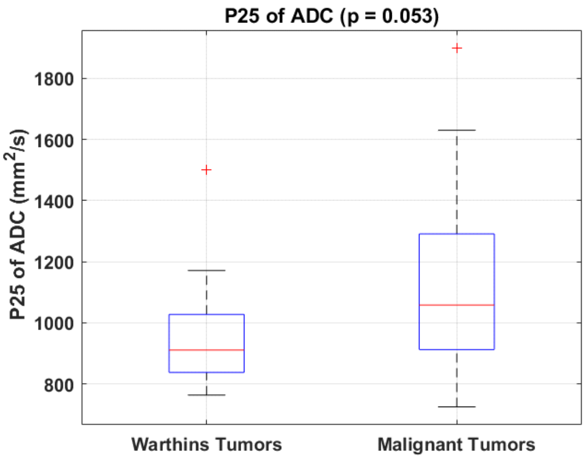


a

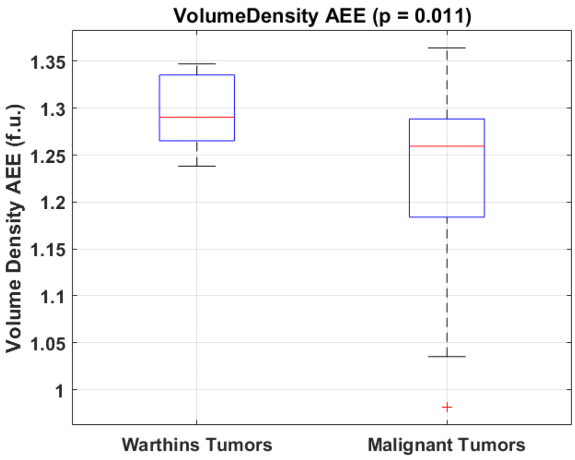


b

**Figure 2.** Box-and-whisker plots of the three features included in the predictive model for discriminating benign versus Warthins' tumors: P25 of ADC (a), Volume Density AEE(Approximate Enclosing Ellipsoid) (b) and the Minimum Histogram Gradient (c) from T2-w images. P-values refer to the Mann-Whitney test.

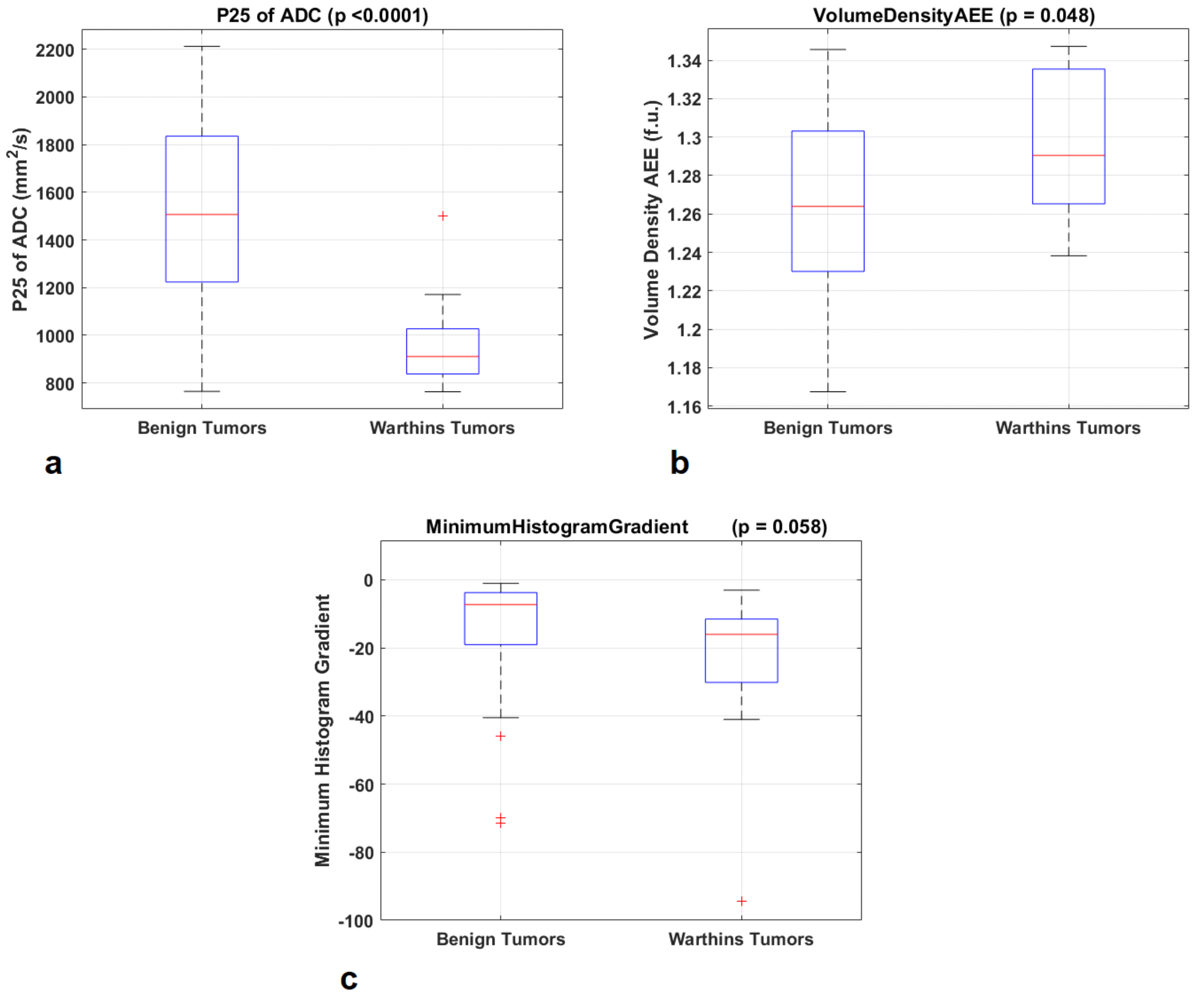

**Figure 3.** Box-and-whisker plots of P25 of ADC and P10 of T2-w signal included in the predictive model for discriminating benign versus malignant tumors. P-value refers to the Mann-Whitney test.

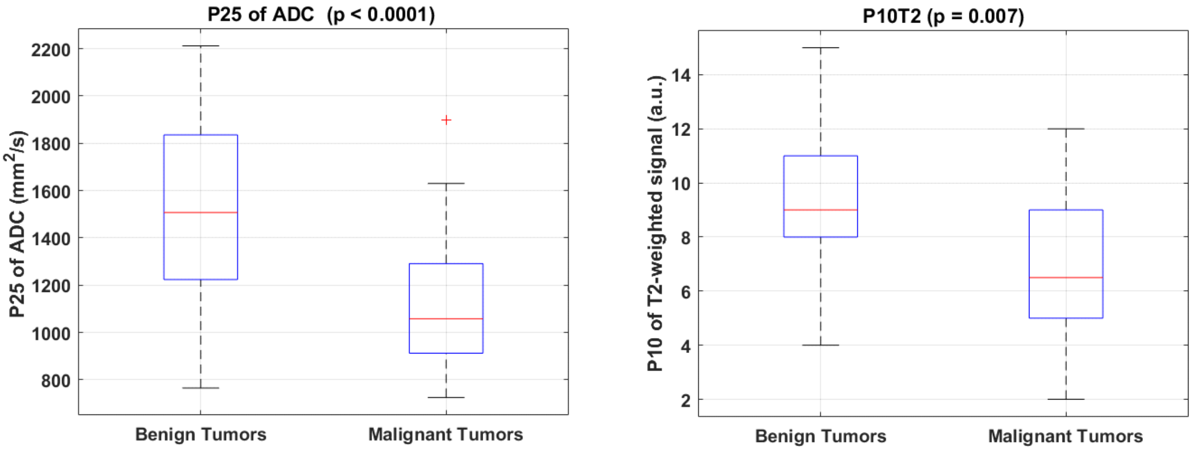


a b
